# Oxidative DNA damage and DNA repair gene variants in differentiated thyroid cancer patients treated with radioactive iodine

**DOI:** 10.1007/s12020-025-04288-7

**Published:** 2025-05-26

**Authors:** Fevzi Necati Avsar, Semra Doğru Abbasoğlu, Evin Ademoğlu, Tuncay Sahutoglu

**Affiliations:** 1 Department of Internal Medicine, Istanbul Faculty of Medicine, Istanbul University, İstanbul, Türkiye; 2 Department of Medical Biochemistry, Istanbul Faculty of Medicine, Istanbul University, İstanbul, Türkiye; 3 Division of Nephrology, Department of Internal Medicine, Mehmet Akif Inan Training and Research Hospital, University of Health Sciences, Sanliurfa, Turkey

**Keywords:** Oxidative DNA damage, Differentiated thyroid cancer, Radioactive iodine therapy, 8-oxoG, HOGG1, APE-1

## Abstract

**Purpose:**

This study aimed to evaluate oxidative DNA damage and its association with polymorphisms in DNA repair enzymes among patients with differentiated thyroid cancer (DTC) treated with high-dose radioactive iodine (RAI, ≥100 mCi). Relationships between DNA damage markers, gene variants, and clinical or tumor characteristics were also explored.

**Methods:**

Seventy-nine patients with DTC and 59 age and sex matched controls from a Turkish population were included. Urinary 8-hydroxy-2′-deoxyguanosine (8-oxoG), a marker of oxidative DNA damage, was measured by ELISA at baseline, 2 weeks, and 3 months post-RAI. Genotyping for hOGG1 Ser326Cys and APE1 Asp148Glu polymorphisms was performed on leukocyte-derived DNA using real-time PCR and melting curve analysis.

**Results:**

Urinary 8-oxoG levels showed a non-significant upward trend over time (*p* = 0.252). The Cys/Cys genotype of hOGG1 was more frequent in patients than controls (13 vs. 6%, *p* = 0.54), while the Ser/Cys genotype was significantly less frequent in patients (36.7 vs. 57.6%; OR: 0.46, 95% CI: 0.22–0.94, *p* = 0.03). Cys allele frequencies were 0.32 in patients and 0.35 in controls. APE1 genotype and allele distributions did not differ significantly between groups. No associations were found between polymorphisms and 8-oxoG levels or clinical features, including DTC subtype, tumor stage, sex, smoking status, or age.

**Conclusion:**

This is the first study to jointly evaluate urinary 8-oxoG and hOGG1/APE1 polymorphisms in Turkish DTC patients receiving RAI. No consistent associations were found with oxidative DNA damage or clinical characteristics. Larger studies are needed to validate these findings.

## Introduction

Differentiated thyroid cancer (DTC), primarily comprising papillary and follicular subtypes, is the most common endocrine malignancy, with its global incidence steadily rising in recent decades. Standard treatment includes total thyroidectomy followed by radioactive iodine (RAI) therapy, leveraging the thyroid tissue’s unique ability to concentrate iodine via the sodium-iodide symporter [1]. While RAI is highly effective in ablating residual or metastatic thyroid tissue, the ionizing radiation it emits can induce reactive oxygen species (ROS), leading to oxidative stress and potential genotoxic effects [2].

ROS can damage DNA through base modifications, strand breaks, and abasic sites, with 8-hydroxy-2′-deoxyguanosine (8-oxoG) being a widely recognized and quantifiable marker of oxidative DNA damage. Unrepaired 8-oxoG lesions can result in G:C to T:A transversions, which contribute to genomic instability, mutagenesis, and cancer development [2]. The base excision repair (BER) pathway plays a pivotal role in the removal of such lesions, with enzymes like human 8-oxoguanine DNA glycosylase (hOGG1) and apurinic/apyrimidinic endonuclease 1 (APE1) acting sequentially to excise oxidized bases and repair apurinic sites [3–5].

Polymorphic variants in these DNA repair genes specifically hOGG1 Ser326Cys and APE1 Asp148Glu may alter enzyme structure or function, modulating the efficiency of BER and potentially influencing individual susceptibility to various cancers [6–10]. These associations have been explored in breast, lung, and colorectal cancer, with inconsistent results that appear to vary by ethnicity, environmental exposures, and genetic background [11, 12]. In thyroid cancers specifically, differential patterns of oxidative DNA damage and repair activity have been documented between histological subtypes, suggesting that DNA repair mechanisms may play subtype-specific roles in tumorigenesis [13].

Despite the extensive literature on RAI-induced genotoxicity, longitudinal assessments of oxidative DNA damage in DTC patients remain limited. Urinary 8-oxoG is a non-invasive, dynamic biomarker that reflects systemic oxidative DNA damage, yet few studies have examined its post-RAI kinetics or its relationship with individual DNA repair capacity. Furthermore, the interaction between 8-oxoG levels and functionally relevant BER gene polymorphisms in DTC patients is poorly understood, particularly in genetically distinct populations such as the Turkish cohort.

This study aims to longitudinally evaluate urinary 8-oxoG levels in DTC patients receiving high-dose RAI therapy and to assess associations with hOGG1 Ser326Cys and APE1 Asp148Glu polymorphisms. By addressing these relationships in a Turkish population, this research seeks to enhance our understanding of how genetic variation influences oxidative stress responses and DNA repair efficacy in the context of thyroid cancer management.

## Methods

This study included 79 patients with differentiated thyroid cancer (DTC) who received high-dose radioactive iodine therapy (RAI ≥ 100 mCi) at the Department of Internal Medicine, Endocrinology and Metabolic Diseases, Istanbul Faculty of Medicine, between February 2011 and March 2012. Patients with diabetes mellitus, chronic renal or liver disease, malignancies other than thyroid cancer, non-DTC thyroid tumors, low-dose RAI, or incomplete clinical or laboratory records were excluded. The study was approved by the Institutional Clinical Research Ethics Committee. All patients provided written informed consent prior to participation.

At the time of admission for high-dose RAI, patients were informed about the study and included after consent was obtained. Demographic and clinical data including age, sex, tumor size, histopathological subtype, presence of lymph node or distant metastases, TNM staging, treatment modalities, cumulative RAI dose, comorbidities, and smoking status were recorded.

The control group consisted of 59 individuals with similar age, sex, comorbidity, and smoking profiles. Individuals with thyroid disease were excluded from the control group.

### Measurement of oxidative DNA damage

Urine samples were intended to be collected from all 79 patients at three time points: prior to RAI therapy (baseline), at 2 weeks, and at 3 months post-treatment. However, due to financial constraints that arose during the later stages of the study, 8-oxoG analysis could only be completed for 55 patients. All collected samples were first morning voids and were stored at −20 °C until analysis. Urinary levels of 8-hydroxy-2′-deoxyguanosine (8-oxoG), a biomarker of oxidative DNA damage, were quantified using commercially available competitive ELISA kits.

### DNA isolation and genotyping

Peripheral venous blood samples were collected into EDTA tubes. Genomic DNA was extracted from leukocytes using the High Pure PCR Template Preparation Kit (Roche Diagnostics GmbH, Mannheim, Germany) and stored at −80 °C. Genotyping for hOGG1 Ser326Cys and APE-1 Asp148Glu polymorphisms was performed using capillary real-time PCR with melting curve analysis and fluorescent hybridization probes on the Light Cycler platform (Roche Diagnostics GmbH, Mannheim, Germany) (Table 1).

**Table 1:** Primer and probe sequences used for genotyping

Target gene (Genbank accession no.) Amino acid change	Primer and probe sequences
APE/Ref-1 (M92444), Asp148Glu	F: 5′-CTTGATTGCTTTCCCTTTTCTTA-3′ R: 5′-CGCTGCCGGTACTCCA-3′ S: 5′LCRed640TGCTCCTCCTCGCCTATAGAAATGAp3′ A: 5′CACAATCACCCGGCCTTCCTGATC-FITC-3′
hOGG1(rs1052133) Ser326Cys	F: 5′-CCCAACACTGTCACTAGTCTCA-3′ R: 5′-TTGGGGAATTTCTTTGTCCA-3′ S: 5′LCRed640CCACCAGCAAAGCGCAGAAAGGGp-3′ A: 5′-CGCCAATCCCGCCATGCTCA

*F* forward primer, *R* reverse primer, *S* sensor prob, *A* anchor prob

### Statistical analysis

Statistical analyses were conducted using SPSS version 15.0 (SPSS Inc., Chicago, IL, USA). Genotype and allele frequencies were compared between patients and controls using the Chi-square test. Odds ratios (ORs) and 95% confidence intervals (CIs) were calculated for genotype comparisons. Genotype distributions in the control group were evaluated for Hardy-Weinberg equilibrium using Pearson’s Chi-square, likelihood ratio (Llr), and exact tests.

The repeated measures ANOVA test was used to compare urinary 8-hydroxy-2′-deoxyguanosine (8-oxoG) levels at three time points: baseline, 2 weeks, and 3 months after radioactive iodine treatment. Descriptive comparisons between clinical variables (e.g., age, sex, smoking) and genotype distributions were made, but no adjusted multivariable analyses were performed. The statistical tests used for these comparisons were not consistently recorded across all analyses and should be interpreted accordingly.

A retrospective sample size calculation was performed using G*Power 3.1.9.7 software to assess the adequacy of the study for repeated measures analysis. Assuming a small-to-moderate effect size (f = 0.15), α = 0.05, and power (1–β) = 0.80, the minimum required sample size was estimated at 73 patients. While urinary 8-oxoG levels could only be measured in 55 patients due to funding constraints, post hoc considerations suggest this sample remains sufficient to detect moderate effect sizes (f = 0.2). As emphasized in the literature, post hoc power assessments have methodological limitations and should not be used to infer the presence or absence of true associations [14, 15].

No correction for multiple comparisons was applied, and the findings should be interpreted with in the exploratory scope of the study.

## Results

The demographic characteristics of the patient and control groups are presented in Tables 2 and 3, respectively.

**Table 2:** Clinical and demographic characteristics of patients with differentiated thyroid cancer (DTC)

Variables	DTC
Age (years)	
Mean ± SD	43.08 ± 13.6
Interval	15–71
Gender	
Female, n (%)	69 (87.3)
Male, n (%)	10 (12.7)
Diagnosis	
Papillary thyroid cancer, n (%)	76 (96.2)
Follicular thyroid cancer, n (%)	2 (2.5)
Mature cystic teratoma, n (%)	1 (1.2)
All group	
Follicular variant, n (%)	46(58.2)
Classical type, n (%)	20 (25.3)
Diffuse sclerosan variant, n (%)	3 (3.7)
Solid variant, n (%)	3 (3.7)
Highly cylindrical variant, n (%)	1 (1.2)
Oncositic variant, n (%)	3 (3.37)
Tumor diameter (cm)	
Mean ± SD	2.17 ± 1.69
Interval	0.4–11.5
Smoking, n (%)	24(30.3)
Radioactive iodine dose(mCi)	
Mean ± SD	141.13 ± 84.2
Interval	100–500

**Table 3:** Clinical and demographic characteristics of the control group

Variables	Control group
Age(year)	
Mean ± SD	45.08 ± 13.6
Interval	20–79
Gender	
Female, n (%)	57 (96.6)
Male, n (%)	2 (3.4)
Smoking, n (%)	15 (25)

The genotype distributions of hOGG1 and APE-1 in the control group were evaluated for Hardy-Weinberg equilibrium (HWE). While the hOGG1 distribution showed borderline deviation (*p* = 0.048), likely due to the small control sample size, APE-1 was in equilibrium (*p* = 0.898).

Genotype and allele frequencies for hOGG1 and APE-1 are summarized in Tables 4 and 5. The frequency of the hOGG1 codon 326 Cys allele was 0.35 in controls and 0.32 in patients, consistent with previously reported frequencies in Turkish populations (e.g., 0.30 in Tanrıkulu et al. [7]). TheCys/Cys genotype was observed in 13% of patients and 6% of controls (*p* = 0.54). The Ser/Cys genotype was significantly less frequent in patients (36.7%) than in controls (57.6%), yielding an odds ratio of 0.46 (95% CI: 0.22–0.94, *p* = 0.03) (Table 4).

**Table 4:** Distribution of hOGG1 codon 326 genotypes and allele frequencies in patients with differentiated thyroid cancer and controls

hOGG1, code 326	Control n (%)	DTC n (%)	OR (%95 CI)	P
Ser/Ser	21(35.5)	39(49.3)	Reference	–
Ser/Cys	34(57.6)	29(36.7)	0,46(0.22–0.94)	0.03
Cys/Cys	4(6)	11(13)	1.48(0.42–5.22)	0.54
Ser/Cys + Cys/Cys	38(64.4)	40(50.6)	0.56(0.28–1.13)	0.10
Cys allele frequency	0.35	0.32	0.86(0.52–1.43)	0.56

**Table 5:** Distribution of APE1 codon 148 genotypes and allele frequencies in patients with differentiated thyroid cancer and controls

APE-1 Code 148	Control n (%)	DTC n (%)	OR (95% CI)	p
GG	26(44)	30(37.9)	Reference	–
GT	26(44)	41(51.8)	1.36(0.66–2.80)	0.39
TT	7(12)	8(10.1)	0.99(0.31–3.10)	0.98
GT + TT	33(55.9)	49(62)	1.28(0.64–2.55)	0.47
T allele frequency	0.34	0.36	1.10(0.66–1.81)	0.70

For APE-1 codon 148, the T allele frequency was 0.34 in controls and 0.36 in patients. These values align with prior reports in Turkish cohorts, including Doğru-Abbasoğlu et al. [8]. The TT genotype was present in 12% of patients and 10.1% of controls, while GT was found in 44% of patients and 51.8% of controls (*p* = 0.30) (Table 5).

**Fig. 1:**
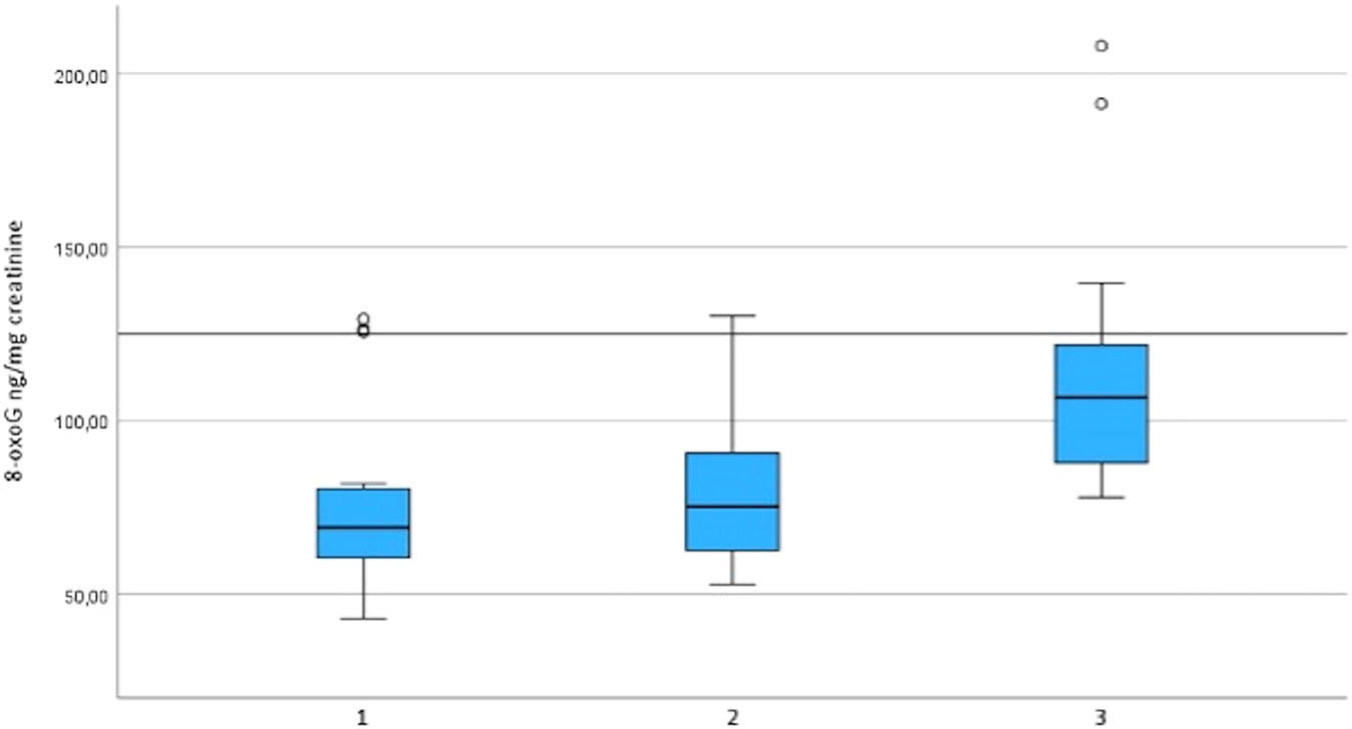
Boxplot graph for urinary 8-hydroxy-2′-deoxyguanosine (8-oxoG) levels (ng/mg creatinine) at baseline (1), 2 weeks (2), and 3 months (3) following radioactive iodine therapy (RAI) in patients with differentiated thyroid cancer (n = 55). No statistically significant differences were observed across time points (repeated measures ANOVA, *p* = 0.251)

Urinary 8-oxoG levels were assessed in 55 patients at baseline, 2 weeks, and 3 months post-RAI using the Friedman test. Although levels showed a slight upward trend, no statistically significant differences were observed across time points (*p* = 0.251; Table 6 and Fig. 1). Additionally, no significant associations were found between 8-oxoG levels and hOGG1 or APE-1 genotypes or alleles.

**Table 6:** Urinary 8-hydroxy-2′-deoxyguanosine (8-oxoG) levels (mean ± SD, median; ng/mg creatinine)

Baseline	73.38 ± 107.72; 35.59
Second week	78.46 ± 100.39; 42.50
Third month	105.37 ± 162.23; 49.20

Clinical parameters such as age, gender, smoking status (pack-years), DTC subtype, tumor stage, and cancer histology (papillary vs. follicular) were analyzed in relation to genotype. No significant correlations were identified between these parameters and hOGG1 or APE-1 genotypes or allelic distributions.

## Discussion

This study examined the relationship between oxidative DNA damage assessed by urinary 8-hydroxy-2′-deoxyguanosine (8-oxoG) and polymorphisms in two key base excision repair enzymes, hOGG1 (Ser326Cys) and APE-1 (Asp148Glu), in patients with differentiated thyroid cancer (DTC) treated with high-dose radioactive iodine (RAI). Although urinary 8-oxoG levels showed a mild increase at 2 weeks and 3 months after RAI, these changes were not statistically significant. Likewise, no consistent associations were observed between either gene polymorphism and urinary 8-oxoG levels or clinical parameters. The Ser/Cys genotype of hOGG1 was significantly less frequent in patients than in controls, suggesting a possible protective association.

8-oxoG is a well-established marker of oxidative DNA damage, most induced by reactive oxygen species and ionizing radiation [2, 16]. If unrepaired, it can lead to mutagenic G:C toT:A transversions and accelerate cellular aging [2, 16]. Chronic accumulation of 8-oxoG in telomeric DNA has also been implicated in telomere shortening and genome instability [17].

Although the observed increase in urinary 8-oxoG levels post-RAI did not reach statistical significance, the trend toward higher levels at 3 months may reflect a delayed or cumulative oxidative stress response. This could indicate either a lag in the generation of oxidative lesions following high-dose RAI or a gradual decline in repair efficiency over time. Alternatively, it may suggest that short-term DNA repair mechanisms, particularly those involving hOGG1 and APE-1, initially buffer oxidative insult but may not fully prevent persistent damage in all patients. Further studies incorporating extended follow-up, additional oxidative stress markers, and functional repair assays are needed to determine whether these observed changes carry long-term clinical significance.

The hOGG1 and APE-1 enzymes are central to the base excision repair (BER) pathway and are responsible for the excision of oxidative DNA lesions such as 8-oxoG [2]. The Ser326Cys substitution in hOGG1 introduces a cysteine residue with a thiol group that could alter the enzyme’s conformation or function [18]. Similarly, phosphorylation-dependent regulation of the serine residue may be disrupted, potentially impairing repair efficiency. Previous studies have demonstrated the relevance of these polymorphisms in various cancers, within consistent findings [12, 13, 17, 19–23]. While our study found a lower frequency of the Ser/Cys genotype in DTC patients compared to controls, this association did not extend to 8-oxoG levels, and theCys/Cys genotype was not significantly different between groups.

Animal and cellular models have provided mechanistic insights into the effects of BER deficiencies. Fouquerel et al. showed that 8-oxoG accumulation in telomeric DNA due to OGG1 loss leads to replication stress and chromosomal instability [17]. Similarly, Ohno et al. reported shortened lifespan and increased tumor incidence in mice lacking Mth1, Ogg1, and Mutyh, emphasizing the importance of these repair enzymes in maintaining genome integrity [21]. However, in contrast to these models, our findings suggest that in clinical settings involving RAI-treated DTC patients, oxidative DNA damage is relatively modest and may not overwhelm the BER capacity in the short term.

For APE-1 Asp148Glu, our results align with previous reports by Chiang et al. and Doğru-Abbasoğlu et al., which found no significant associations between this polymorphism and thyroid-related disease risk [20, 22]. Similarly, in our cohort, genotype distributions and urinary 8-oxoG levels did not differ significantly between patients and controls.

We also analyzed clinical and pathological parameters including tumor subtype, stage, gender, age, and smoking habits in relation to genotype and 8-oxoG levels. No significant associations were identified, suggesting that neither polymorphism is linked to disease phenotype or oxidative stress burden in this population.

Although a statistically significant association was observed for the hOGG1 Ser/Cys genotype, this finding should be interpreted with caution. The study’s modest sample size and lack of covariate-adjusted models limit the strength of inference. A post hoc power analysis was planned but not reported in detail, and such analyses have limited interpretive value [14, 15]. Furthermore, correction for multiple testing was not applied, and the potential for false-positive results must be considered. Future research with larger, more diverse cohorts and multivariable modeling is warranted to confirm these associations and clarify the biological role of BER variants in RAI-treated DTC patients.

This study’s strengths include the longitudinal design with repeated measurement of urinary 8-oxoG, the use of validated genotyping methods for functionally relevant DNA repair gene polymorphisms, and the inclusion of a demographically matched control group. However, limitations must be acknowledged. The sample size may have limited statistical power, particularly for detecting genotype–phenotype associations involving less frequent alleles. Although the study was originally powered to detect small-to-moderate effects and enrolled enough patients (n = 79), urinary 8-oxoG measurements could only be completed in 55 patients due to unforeseen funding constraints following the lead investigator’s illness and passing. Individual 8-oxoG variability and the influence of unmeasured confounders such as diet or renal clearance may also affect biomarker interpretation. Additionally, multivariable adjustment and correction for multiple comparisons were not applied, which should be addressed in future studies.

## Conclusion

To our knowledge, this is the first study to investigate hOGG1 (codon 326) and APE-1 (codon 148) polymorphisms alongside urinary 8-oxoG levels in DTC patients undergoing high-dose RAI therapy. While the findings suggest a possible protective role for the hOGG1 Ser/Cys genotype, no consistent association was observed between these polymorphisms and DTC susceptibility, oxidative DNA damage, or clinical characteristics. Larger studies incorporating broader genetic profiling and functional validation are needed to clarify the role of DNA repair gene variants in thyroid cancer risk.

## Data Availability

No datasets were generated or analysed during the current study.

## References

[1] M. Tulchinsky et al., Radioactive iodine therapy for differentiated thyroid cancer: lessons from confronting controversial literature on risks for secondary malignancy. J. Nucl. Med. **59**(no. 5), 723–725 (2018). 10.2967/jnumed.118.211359. 29653977 10.2967/jnumed.118.211359

[2] S. Maynard, S.H. Schurman, C. Harboe, N.C. de Souza-Pinto, V.A. Bohr, Base excision repair of oxidative DNA damage and association with cancer and aging. Carcinogenesis **30**(no. 1), 2–10 (2008). 10.1093/carcin/bgn250. 18978338 10.1093/carcin/bgn250 PMC2639036

[3] S. Roychoudhury et al., Endogenous oxidized DNA bases and APE1 regulate the formation of G-quadruplex structures in the genome. Proc. Natl Acad. Sci. **117**(no. 21), 11409–11420 (2020). 10.1073/pnas.1912355117. 32404420 10.1073/pnas.1912355117 PMC7260947

[4] S. Pramanik, Y. Chen, K.K. Bhakat, Base excision repair in mitotic cells and the role of apurinic/apyrimidinic endonuclease 1 (APE1) in post-mitotic transcriptional reactivation of genes. Int. J. Mol. Sci. **25**(no. 23), 12735 (2024). 10.3390/ijms252312735. 39684445 10.3390/ijms252312735 PMC11641725

[5] J. Sung, B. Demple, Roles of base excision repair subpathways in correcting oxidized abasic sites in DNA. FEBS J. **273**(no. 8), 1620–1629 (2006). 10.1111/j.1742-4658.2006.05192.x. 16623699 10.1111/j.1742-4658.2006.05192.x

[6] Y. Shi, W. Xu, X. Zhang, Association of the hOGG1 Ser326Cys polymorphism with gynecologic cancer susceptibility: a meta-analysis. Biosci. Rep. **40**(no. 12), BSR20203245 (2020). 10.1042/BSR20203245. 33210702 10.1042/BSR20203245 PMC7693197

[7] D. Zhong, G. Li, J. Long, J. Wu, Y. Hu, The hOGG1Ser326Cys polymorphism and increased lung cancer susceptibility in caucasians: an updated meta-analysis. Sci. Rep. **2**(no. 1), 548 (2012). 10.1038/srep00548. 22855704 10.1038/srep00548 PMC3409380

[8] F. AlMutairi et al., Association of DNA repair gene APE1 Asp148Glu polymorphism with breast cancer risk. Dis. Markers **2015**, 1–10 (2015). 10.1155/2015/869512. 10.1155/2015/869512 PMC4519542 26257461

[9] C. Liu, Q. Yin, L. Li, Y. Zhuang, X. Zu, Y. Wang, APE1 Asp148Glu gene polymorphism and bladder cancer risk: a meta-analysis. Mol. Biol. Rep. **40**(no. 1), 171–176 (2013). 10.1007/s11033-012-2046-5. 23143180 10.1007/s11033-012-2046-5

[10] Y.-L. Lo et al., A polymorphism in the APE1 gene promoter is associated with lung cancer risk. Cancer Epidemiol. Biomark. Prev. **18**(no. 1), 223–229 (2009). 10.1158/1055-9965.EPI-08-0749. 10.1158/1055-9965.EPI-08-0749 19124501

[11] E. Canbay et al., Association of APE1 and hOGG1 polymorphisms with colorectal cancer risk in a Turkish population. Curr. Med. Res. Opin. **27**(no. 7), 1295–1302 (2011). 10.1185/03007995.2011.573544. 21561390 10.1185/03007995.2011.573544

[12] X. Xue et al., The Joint Effect of hOGG1, APE1, and ADPRT polymorphisms and cooking oil fumes on the risk of lung adenocarcinoma in chinese non-smoking females. PLoS One **8**(no. 8), e71157 (2013). 10.1371/journal.pone.0071157. 23951099 10.1371/journal.pone.0071157 PMC3741325

[13] S. Karger et al., Distinct pattern of oxidative DNA damage and DNA repair in follicular thyroid tumours. J. Mol. Endocrinol. **48**(no. 3), 193–202 (2012). 10.1530/JME-11-0119. 22331172 10.1530/JME-11-0119

[14] C. Andrade, Sample size and its importance in research. Indian. J. Psychol. Med. **42**(no. 1), 102–103 (2020). 10.4103/IJPSYM.IJPSYM_504_19. 31997873 10.4103/IJPSYM.IJPSYM_504_19 PMC6970301

[15] S. Lydersen, Statistical power: before, but not after!, *Tidsskr. Nor. Laegeforen*., 139, no. 2, 2019, 10.4045/tidsskr.18.0847. 10.4045/tidsskr.18.0847 30698397

[16] N. Watanabe et al., Radiotoxicity after iodine-131 therapy for thyroid cancer using the micronucleus assay. *J. Nucl. Med.* **39**(no. 3), 436–440 (1998). http://www.ncbi.nlm.nih.gov/pubmed/9529288. 9529288

[17] E. Fouquerel, R.P. Barnes, S. Uttam, S.C. Watkins, M.P. Bruchez, P.L. Opresko, Targeted and persistent 8-oxoguanine base damage at telomeres promotes telomere loss and crisis. Mol. Cell **75**(no. 1), 117–130.e6 (2019). 10.1016/j.molcel.2019.04.024. 31101499 10.1016/j.molcel.2019.04.024 PMC6625854

[18] M.P. Kaur, E.J. Guggenheim, C. Pulisciano, S. Akbar, R.M. Kershaw, N.J. Hodges, Cellular accumulation of Cys326-OGG1 protein complexes under conditions of oxidative stress. Biochem. Biophys. Res. Commun. **447**(no. 1), 12–18 (2014). 10.1016/j.bbrc.2014.03.044. 24680828 10.1016/j.bbrc.2014.03.044 PMC4005915

[19] S. Tanrıkulu et al., The 8-oxoguanine DNA N -glycosylase 1 (hOGG1) Ser326Cys variant affects the susceptibility to Graves’ disease. Cell Biochem. Funct. **29**(no. 3), 244–248 (2011). 10.1002/cbf.1742. 21465496 10.1002/cbf.1742

[20] S. Doğru-Abbasoğlu et al., Polymorphisms of DNA base-excision repair genes APE/Ref-1 and XRCC1 are not associated with the risk for Graves’ disease. Cell Biochem. Funct. **27**(no. 7), 462–467 (2009). 10.1002/cbf.1595. 19711438 10.1002/cbf.1595

[21] M. Ohno et al., 8-oxoguanine causes spontaneous de novo germline mutations in mice. Sci. Rep. **4**(no. 1), 4689 (2014). 10.1038/srep04689. 24732879 10.1038/srep04689 PMC3986730

[22] F.-Y. Chiang et al., Association between polymorphisms in DNA base excision repair genes XRCC1, APE1, and ADPRT and differentiated thyroid carcinoma. Clin. Cancer Res. **14**(no. 18), 5919–5924 (2008). 10.1158/1078-0432.CCR-08-0906. 18779313 10.1158/1078-0432.CCR-08-0906

[23] A. Elahi, The human OGG1 DNA repair enzyme and its association with orolaryngeal cancer risk. Carcinogenesis **23**(no. 7), 1229–1234 (2002). 10.1093/carcin/23.7.1229. 12117782 10.1093/carcin/23.7.1229

